# Social Behavior of Antibiotic Resistant Mutants Within *Pseudomonas aeruginosa* Biofilm Communities

**DOI:** 10.3389/fmicb.2019.00570

**Published:** 2019-03-22

**Authors:** Estrella Rojo-Molinero, María D. Macià, Antonio Oliver

**Affiliations:** Servicio de Microbiología, Hospital Son Espases, Instituto de Investigación Sanitaria Illes Balears (IdISBa), Palma de Mallorca, Spain

**Keywords:** *Pseudomonas aeruginosa*, biofilms, bacterial communities, resistance mutants, β-lactam antibiotics, antibiotic resistance

## Abstract

The complex spatial structure and the heterogeneity within biofilms lead to the emergence of specific social behaviors. However, the impact of resistant mutants within bacterial communities is still mostly unknown. Thus, we determined whether antibiotic resistant mutants display selfish or altruistic behaviors in mixed *Pseudomonas aeruginosa* biofilms exposed to antibiotics. ECFP-tagged *P. aeruginosa* strain PAO1 and its EYFP-tagged derivatives hyperproducing the β-lactamase AmpC or the efflux pump MexAB-OprM were used to develop single or mixed biofilms. Mature biofilms were challenged with different concentrations of β-lactams to monitor biofilm structural dynamics, using confocal laser scanning microscopy (CLSM), and population dynamics, through enumeration of viable cells. While exposure of single wild-type PAO1 biofilms to β-lactams lead to a major reduction in bacterial load, it had little effect on biofilms formed by the resistant mutants. However, the most reveling finding was that bacterial load of wild-type PAO1 was significantly increased when growing in mixed biofilms compared to single biofilms. In agreement with CFU enumeration data, CLSM images revealed the amplification of the resistant mutants and their protection of susceptible populations. These findings show that mutants expressing diverse resistance mechanisms, including β-lactamases, but also, as evidenced for the first time, efflux pumps, protect the whole biofilm community, preserving susceptible populations from the effect of antibiotics. Thus, these results are a step forward to understanding antibiotic resistance dynamics in biofilms, as well as the population biology of bacterial pathogens in chronic infections, where the coexistence of susceptible and resistant variants is a hallmark.

## Introduction

Biofilms are organized bacterial communities embedded in an extracellular polymeric matrix, often attached to living or abiotic surfaces. The development of biofilms is currently recognized as one of the most frequent modes of growth in the environment and the main driver of persistence of chronic infections. Furthermore, characteristically, these bacterial communities are highly resistant to the immune system and antibiotics (Costerton et al., 1999). The increased tolerance to antibiotics is multi-factorial and it is attributed to a restricted penetration of the antimicrobial molecules, limited growth at low oxygen concentration, the expression of biofilm-specific genes and the presence of dormant or persister cells (Høiby et al., 2010; Stewart, 2015). However, in addition to the physical and physiological tolerance, classical resistance mechanisms play a major role in biofilm antibiotic resistance (Høiby et al., 2010). Over the past few years many works have been focused on the effect of antimicrobial resistance mechanisms on planktonic mode of growth, but there is still a lack of knowledge about the impact of these resistance mechanisms on bacterial communities such as biofilms.

Moreover, it should be noted that bacterial communities are not usually formed by a single strain. Thereby, within biofilms, genotypic variants of an isogenic strain, different strains and even different species can be found. This heterogeneity along with the complex spatial structure of biofilm colonies lead to cell-to-cell interactions and the emergence of social behaviors of cooperation and competition (Nadell et al., 2016).

One of the clearest examples of cooperative phenomenon in bacterial communities is the antibiotic inactivation by the action of enzymes such as the β-lactamase AmpC. Some studies have demonstrated that the release of β-lactamases into the matrix appears to protect biofilms against harmful β-lactams showing that it plays a major role in biofilm resistance (Bagge et al., 2004a,b; Mulet et al., 2011). Likewise, some works have shown that cooperative or altruist behaviors are promoted in biofilms whereas in batch cultures selection of the fittest is favored, like the selection of resistant mutants during antibiotic treatment at the expense of susceptible cells (Kreft, 2004; Frost et al., 2018).

On the other hand, there is still no specific data regarding the effect of other resistance mechanisms, especially for those that deal with antibiotic concentration inside the biofilm, such as the expression of efflux pumps. Hypothetically, while the permeation of the β-lactamase AmpC into the biofilm matrix by mutants overexpressing AmpC during β-lactam treatment should be beneficial for the whole community, the extrusion of antibiotic by mutants overexpressing an efflux pump, even if it were advantageous at individual cell level, could entail a collective damage for the general susceptible biofilm population by the subsequent accumulation of the antibiotic in the matrix. Therefore, the aim of the present study was to determine whether mutants with different antibiotic resistance mechanisms display an altruistic (cooperative) or selfish behavior in mixed biofilm communities exposed to antibiotic treatments and how these behaviors have an impact on the structure and population dynamics.

For these purposes, the opportunistic pathogen *Pseudomonas aeruginosa*, a paradigm of biofilm formation and chronic infections (such as chronic respiratory infections in cystic fibrosis patients), was chosen to carry out the experiments. The most relevant *P. aeruginosa* mutational resistance mechanisms are those leading to overexpression of the chromosomal β-lactamase AmpC together with the inactivation of porins, or the upregulation of several efflux pumps encoded in its elastic genome. Specifically, the overexpression of AmpC is frequently triggered by the inactivation of the non-essential penicillin-binding protein PBP4, encoded *dacB* gene (Moyà et al., 2009) and confers high-level resistance to antipseudomonal penicillins and cephalosporins. Another mechanism that confers resistance to β-lactam antibiotics is the overexpression of the MexAB-OprM efflux pump mainly due to the inactivation of its negative regulator *mexR* (Poole et al., 1996). Thus, we constructed *mexR* and *dacB* knockout mutants, mixed each of them with a susceptible strain and subjected the mixtures to different treatments with β-lactam antibiotics, both in biofilm and planktonic experiments. Moreover, we used an *in vitro* flow cell biofilm model, an open system that attempts to replicate the *in vivo* conditions through the control of nutrient delivery, flow, and temperature. This model allows the application of pharmacokinetic/pharmacodynamic (PK/PD) parameters, as well as an *in situ* and non-destructive follow-up of the structural dynamics of biofilms under treatments through the use of confocal laser scanning microscopy (CLSM).

## Materials and Methods

### Strains, Plasmids, and Construction of Knockout Mutants

The strains used in this study are listed in Table 1. The wild-type, piliated, reference strain PAO1, was obtained from the Danish collection (Systems Biology-DTU) (Holloway, 1955; Heydorn et al., 2000). PAO1 strain was previously tagged at the *att* intergenic neutral chromosomal locus with ECFP (cyan fluorescent protein) or EYFP (yellow fluorescent protein) in mini-Tn7 constructs containing gentamicin (10 mg/l) and streptomycin (200 mg/l) (Macià et al., 2011), respectively, as described by Klausen et al. (2003).

**Table 1:** Strains and plasmids used in this work.

Strain or plasmid	Genotype or relevant characteristic(s)	Reference
**Strains**		
***P. aeruginosa***		
PAO1	Reference strain completely sequenced *Cfp* fluorescently tagged containing gentamicin (10 mg/L) as resistance marker *Yfp* fluorescently tagged containing streptomycin (200 mg/L) as resistance marker	Heydorn et al., 2000; Macià et al., 2011
PAOΔ*dacB*	PAOYFPΔ*dacB::lox dacB* encodes the non-essential Penicillin Binding Protein 4 (PBP4) β-lactamase AmpC hyperproducer	This work
PAOΔ*mexR*	PAOYFPΔ*mexR::lox mexR* is a repressor of the *mexAB-OprM* multidrug efflux operon Efflux pump MexAB-OprM hyperproducer	This work
***E. coli***		
S17.1	RecA pro (RP4-2Tet::Mu Kan::Tn7)	Laboratory collection
**Plasmids**		
pEXdacBGm	pEX100Tlink containing 5–3 flanking sequence of dacB::Gmlox	Moyà et al., 2009
pEXMexRGm	pEX100Tlink containing 5′ and 3′ flanking sequence of mexR::Gmlox	Mulet et al., 2011

PAO1 EYFP-tagged single knockout mutants were constructed following the adapted conditions from the protocol described by Moyà et al. (2009) based on the *Cre-lox* system for gene deletion and antibiotic resistance marker recycling. Previously constructed plasmids (pEXTΔ*dacB*::Gm and pEXTΔ*mexR*::Gm) were used for the generation of *dacB* and *mexR* mutants (Moyà et al., 2009; Mulet et al., 2011). Plasmids were then transformed into the *Escherichia coli* S17-1 helper strain. Knockout mutants were generated by conjugation followed by selection of double recombinants by the use of sucrose (5%)-cefotaxime (1 mg/l)-gentamicin (30 mg/l)-LB agar plates. Double recombinants were checked by screening for carbenicillin (250 mg/l) susceptibility and checked afterward by PCR amplification. To remove gentamicin resistance cassettes, plasmids pCM157 were transformed by electroporation into the mutants. Transformed colonies were checked by plating in LB agar supplemented with tetracycline (250 mg/l). One transformed colony of each mutant was grown overnight in 250 mg/l tetracycline LB broth to allow expression of the *cre* recombinase. Plasmid pCM157 was then cured from the mutants by successive passages on LB broth. Thereafter, selected colonies were screened for tetracycline (250 mg/l) and gentamicin (30 mg/l) susceptibility and checked by PCR amplification.

### Antibiotic Susceptibility Testing

In order to select the most adequate antibiotics for the experiments, minimal inhibitory concentrations (MICs) of ticarcillin, piperacillin-tazobactam, ceftazidime, cefepime (FEP), aztreonam (ATM), ceftolozane-tazobactam, imipenem, meropenem, ciprofloxacin, tobramycin, amikacin, and colistin were determined by broth microdilution (Thermo Scientific Sensititre ID/AST System) for each fluorescently labeled strain following the standard procedures. The MICs were reported after 24 h of incubation at 37°C. EUCAST breakpoints were used^1^.

### Motility Assays

#### Swimming Motility

Isolated colonies from an overnight culture in LB agar were inoculated with a sharp sterile toothpick in swimming medium (10 g/l tryptone, 5 g/l NaCl, and 0.3% [wt/vol] midresolution agarose) plates (Rashid and Kornberg, 2000).

#### Swarming Motility

Swarm agar (M8 minimal medium supplemented with 1 mM MgSO4, 0.2% glucose, 0.5% Bacto Casamino Acids, and 0.5% agar) plates were spotted using 2.5 μl from overnight LB broth cultures (Caiazza et al., 2005).

#### Twitching Motility

With a sharp sterile toothpick isolated colonies from an overnight culture were inserted to the bottom of LB agar plates (Rashid and Kornberg, 2000).

All the motility assays were carried out in triplicate. After 16 h of incubation at 37°C, the zone of motility was measured. If the area to be measured was irregular, three perpendicular diameters were measured and the result was expressed as the mean of the three values.

### Growth Rates

Doubling time for each strain were determined by plating, at 1.5 h intervals, serial 10-fold dilutions of exponentially growing cells in LB broth at 37°C and 180 rpm on LB Agar. As well, a growth curve was constructed for each strain by monitoring at 30 min interval the optical density at 600 nm until reaching stationary phase. Three independent experiments for each strain were performed.

### Flow Cell Model for Biofilm Treatment

Biofilms were grown at 30°C in three-channel flow cells (individual channel dimensions of 1 by 4 by 40 mm) supplied with modified FAB medium (Heydorn et al., 2000) supplemented with 0.3 mM glucose. The flow system was assembled and prepared as described previously (Christensen et al., 1999). Channels were inoculated with 250 μl of normalized dilutions (1/100 dilution of cultures adjusted to an optical density at 600 nm of 0.1) of saturated bacterial cultures and left without flow for 1 h to allow bacterial adherence. A medium flow was then started at a constant rate of 3 ml ⋅ h^-1^ using a Watson-Marlow 205S peristaltic pump.

After 48 h (time 0 [t0]), biofilms were challenged with FEP at 1, 2, or 4 mg/l for 6 days. FEP concentrations correlated to one, two, and fourfold PAO1 MIC. The antibiotic was added to fresh medium and renewed daily. Untreated biofilms were also studied as control biofilms.

At time point 6 (6 days of treatment or 8-day-old biofilm, [t6]), biofilms were detached and collected by washing the flow cell channels with 1 ml of glass beads (Sigma) suspension in 0.9% NaCl. Viable cells were determined for treated and control biofilms for each strain. For this purpose, serial 1/10 dilutions of the suspensions were plated in MHA supplemented with 10 mg/l of gentamicin or 200 mg/l of streptomycin to determine the CFU numbers of PAO1 and its knockout mutants (PAOΔ*dacB* or PAOΔ*mexR*), respectively. The results from at least three independent experiments were considered.

### Microscopic Analysis

Biofilm structural dynamics were monitored by CLSM at time points 0, 2, 4, and 6. All microscopic observations were performed by using a Zeiss LSM710 CLSM (Carl Zeiss, Jena, Germany) equipped with a multiline argon laser, detector, and filter sets for monitoring ECFP (excitation 434 nm, emission 474 nm) and EYFP expression (excitation 514 nm, emission 527 nm). Images were obtained by using 63×/1.4 oil Plan Apo objective lenses. For the study of structural dynamics of biofilms during treatment, at least four pictures per channel, flow cell and strain were taken at t6. Simulated three-dimensional (3D) images and sections were generated by using the IMARIS software package (Bitplane AG, Zurich, Switzerland).

### Competition Experiments in Biofilms

Competition experiments between PAO1 (ECFP-tagged) and its knockout mutants PAOΔ*dacB* or PAOΔ*mexR* (EYFP-tagged) were started at 1:0.01 and 1:1 proportions. Similar to individual biofilms, after 48 h of incubation (t0), mixed biofilms were challenged with FEP at 1, 2, or 4 mg/l for 6 days (t6). 1:0.01 initial ratio experiments were only performed with FEP at 2 mg/l. Biofilms were monitored by CLSM over time and pictures were taken at t6. Biofilms were detached, collected and plated as described above to determine the CFU numbers of PAO1 and its mutants (PAOΔ*dacB* or PAOΔ*mexR*). Untreated biofilms controls were also studied. The results from at least three independent experiments were considered.

### Experiments on Planktonic Cells

Overnight cultures of PAO1 and resistant mutants were used to inoculate 10^6^ CFU/ml in fresh modified FAB medium (Heydorn et al., 2000) supplemented with 0.3 mM glucose and 1, 2, or 4 mg/l of FEP. Single strain stationary cultures and 1:1 initial ratio competitions were performed. Untreated cultures were also studied as controls. The cultures were left to grow at 37°C and 180 rpm for 24 h. As in biofilm experiments, CFU numbers of PAO1 and mutants were determined by plating serial 1/10 dilutions of the cultures in MHA supplemented with antibiotics (see above). The results from at least three independent experiments were considered. Additional studies using a 10^8^ CFU/ml initial inoculum were performed with PAO1 and 1:1 competitions with all FEP concentrations.

### Statistical Analysis

Bacterial load data of each strain of single versus competition for both planktonic and biofilms experiments, as well as, the growth rates and motility parameters for each of the mutants were compared with those for PAO1 using a Student’s *t*-test. A *P*-value of <0.05 was considered statistically significant.

## Results

### Susceptibility Testing and Characterization of the Strains

In order to choose the most suitable treatment for the experiments, several antibiotics were tested to define the susceptibility profile of all fluorescently labeled strains (Table 2). As expected from previous works (Poole et al., 1996; Moyà et al., 2009), AmpC hyperproduction and MexAB-OprM overexpression, driven by *dacB* and *mexR* mutations, significantly increased the MICs of β-lactam antibiotics. Since that the β-lactams FEP and ATM, showed the same increase in the MIC (eightfold respect PAO1) with both resistant mutants, these antibiotics were chosen for the experiments.

**Table 2:** Susceptibility profiles of strains used in this work.

Strain	MIC (mg/l)											
	TIC	P/T	TAZ	FEP	ATM	C/T	IMI	MER	CIP	TOB	AMI	COL
PAO1^a^	8	4/4	1	1	2	0.5/4	4	1	0.25	0.5	4	2
PAOΔ*dacB*^b^	256	64/4	32	8	16	2/4	2	1	0.25	0.5	2	4
PAOΔ*mexR*^b^	128	16/4	4	8	16	1/4	2	2	0.5	0.5	2	4

*^a^ECFP-tagged; ^b^EYFP-tagged; TIC, ticarcillin; P/T, piperacillin-tazobactam; TAZ, ceftazidime; FEP, cefepime; ATM, aztreonam; C/T, ceftolozane-tazobactam; IMI, imipenem; MER, meropenem; CIP, ciprofloxacin; TOB, tobramycin; AMI, amikacin; COL, colistin.*

To rule out if the construction of PAO1 single knockout mutants has an effect on bacterial motility, swimming, swarming and twitching were studied. As it is widely known motility systems, especially swimming (flagellum-mediated motility) and twitching (type IV fimbria-mediated motility) have an important role in the colonization of surface and therefore biofilm development (Jarrell and McBride, 2008). The experiments showed no statistically significant difference between the motility parameters for each resistant mutant compared with PAO1 (Supplementary Figure S1A). Moreover, bacterial growth was not compromise in resistant mutants showing, overlapping curves with PAO1 (Supplementary Figure S1B) and the same doubling times (18.2 min).

### Dynamics of Biofilm Structure and Survival Under β-Lactam Treatment

**FIGURE 1:**
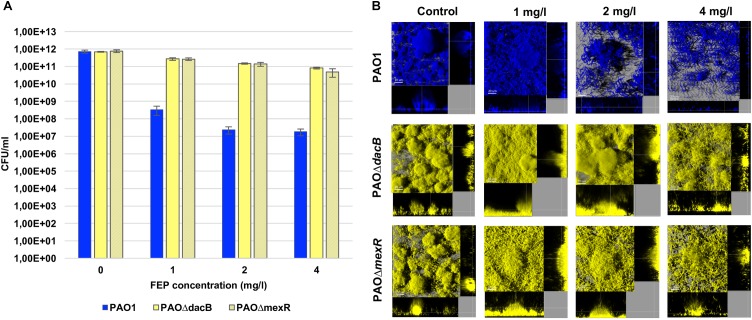
**(A)** Biofilm cell counts (CFU/ml) after 6 days (t6) of treatment with FEP (0, 1, 2, or 4 mg/l). The results represent the means (bars) and standard deviations (error bars) from at least three independent experiments. **(B)** Three-dimensional images and transversal sections of ECFP-tagged (cyan) PAO1 and its EYFP-tagged (yellow) derivative PAOΔdacB, and PAOΔmexR biofilms treated with FEP (0, 1, 2, or 4 mg/l). Images obtained at t6 (8-day-old biofilms, 6 days of FEP treatment) are shown.

Exposure of wild-type PAO1 biofilms to FEP led to a significant reduction in the bacterial load ranging from 3 logs (1 mg/l) to 4 logs (2 and 4 mg/l) (Figure 1A). On the other hand, as could be expected by FEP MIC of resistant mutants (8 mg/l), FEP treatment only had a minor effect on resistant mutants biofilms and, viable cells, even at the highest concentration, remained almost at the same level of those of untreated control biofilm (Figure 1A).

Accordingly, PAO1 CLSM images showed a disruption of biofilm structures along with a strong filamentation effect, which increased proportionally to antibiotic concentration (Figure 1B). Biofilms by resistant mutant strains conserved a mushroom-like structure for all concentrations, despite a slight filamentation was observed in both strains. Although this effect was more obvious at FEP 4 mg/l, PAOΔ*mexR* biofilm cells started to elongate at subinhibitory concentrations (Figure 1B).

### Amplification of Resistant Populations During Antibiotic Treatment

**FIGURE 2:**
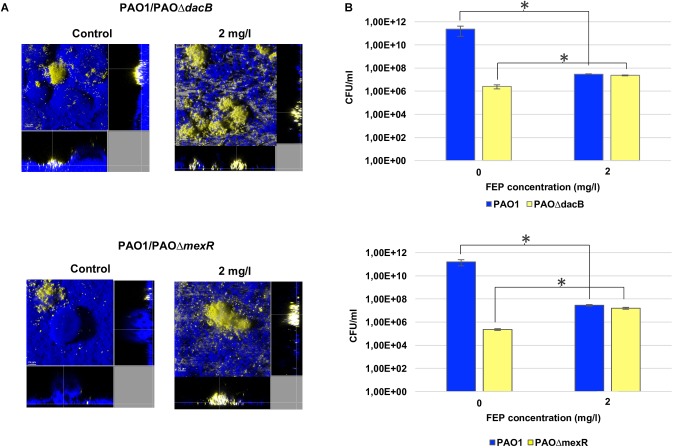
**(A)** Three-dimensional images and transversal sections of competition experiments (1:0.01 initial ratio) between ECFP-tagged (cyan) PAO1 and its EYFP-tagged (yellow) derivative PAOΔdacB or PAOΔmexR treated with FEP (0 or 2 mg/l). Images obtained at t6 (8-day-old biofilms, 6 days of FEP treatment) are shown. **(B)** Cell counts (CFU/ml) of mixed biofilms after 6 days (t6) of treatment with FEP (0 or 2 mg/l). The results represent the means (bars) and standard deviations (error bars) from at least three independent experiments. ^∗^Statistically significant differences between treated and untreated biofilms.

To investigate the dynamics of the selection of resistant mutants in biofilms during treatment, competition experiments between PAO1 and PAOΔ*dacB*, or PAOΔ*mexR*, were initiated at 1:0.01 ratios. Biofilms were exposed to 2 mg/l of FEP, which correspond to twoflod and one/fourfold the MIC of PAO1 and resistant mutants, respectively. The CLSM images (Figure 2A) revealed both PAOΔ*dacB* and PAOΔ*mexR* were able to grow and develop microcolonies after 6 days of treatment with subinhibitory antibiotic concentration, while in untreated control biofilms practically the whole structure is formed by PAO1 strain. In agreement with microscopic observations, biofilms bacterial loads of both resistant strains increased over time almost reaching PAO1 values (Figure 2B). In fact, after 6 days of FEP treatment, the resistant mutant/PAO1 ratio in mixed biofilms suffered a statistically significant increase from 0.01 to 0.77 for PAO1/PAOΔ*dacB*, and to 0.56 for PAO1/PAOΔ*mexR*, while in untreated control biofilms the resistant mutant/PAO1 ratio was 0.0001. These results demonstrated the selection and amplification of antibiotic resistant populations in biofilms during FEP treatment.

### Resistant Mutants Shield Biofilms From Antibiotic Treatment

**FIGURE 3:**
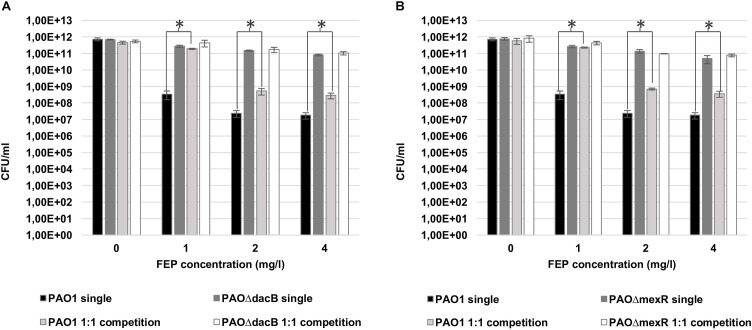
Cell counts (CFU/ml) of PAO1 and knockout mutants (**A**: PAOΔ*dacB*; **B**: PAOΔ*mexR*) in single or mixed biofilms (1:1 competition) after 6 days (t6) of treatment with FEP (0, 1, 2, or 4 mg/l). The results represent the means (bars) and standard deviations (error bars) from at least three independent experiments. ^∗^Statistically significant differences between mixed and single PAO1 biofilms cell counts.

Bacterial loads of both resistant mutants in mixed 1:1 biofilms after antibiotic treatments were similar to those obtained in single biofilms experiments (Figure 3). However, interestingly, a statistically significant increase in PAO1 bacterial load (3 logs with 1 mg/l and 1 log with 2 and 4 mg/l of FEP) was observed when growing in the 1:1 mixed biofilm with each of the resistant mutants compared to the single PAO1 biofilm (Figure 3). No differences in cell counts were observed between PAO1 and the mutants when growing together without antibiotic treatment.

**FIGURE 4:**
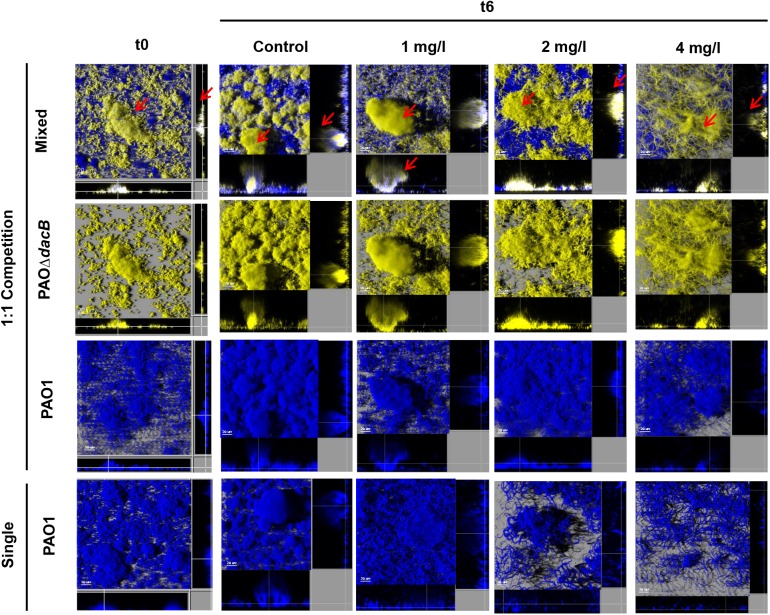
Three-dimensional images and transversal sections of competition experiments (1:1 initial ratio) between ECFP-tagged (cyan) PAO1 and its EYFP-tagged (yellow) derivative PAOΔ*dacB* and ECFP-tagged (cyan) single biofilm treated with FEP (0, 1, 2, or 4 mg/l). Images obtained at t0 and t6 (8-day-old biofilms, 6 days of FEP treatment) are shown. Red arrows mark mutant strain surrounding PAO1.

**FIGURE 5:**
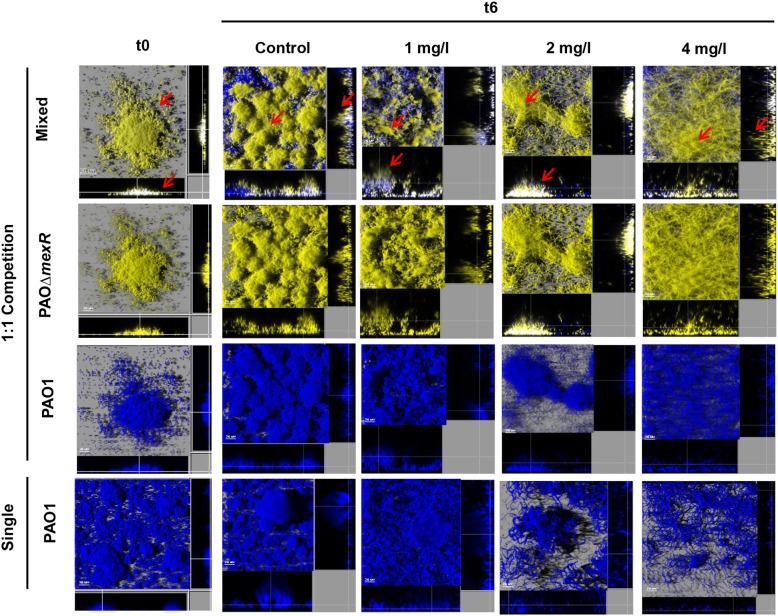
Three-dimensional images and transversal sections of competition experiments (1:1 initial ratio) between ECFP-tagged (cyan) PAO1 and its EYFP-tagged (yellow) derivative PAOΔ*mexR* and ECFP-tagged (cyan) single biofilm treated with FEP (0, 1, 2, or 4 mg/l). Images obtained at t0 and t6 (8-day-old biofilms, 6 days of FEP treatment) are shown. Red arrows mark mutant strain surrounding PAO1.

Likewise, 3D-reconstructed images confirmed a preservation of biofilm structure in PAO1, along with less filamentation, in correlation with CFU counts (Figure 4, 5). Moreover, CLSM images showed how PAOΔ*dacB* and PAOΔ*mexR* surrounded PAO1, located at the outer part of biofilms and thus, likely protecting the susceptible strain from antibiotic. However, this kind of distribution was seen for untreated control mixed biofilms. For that reason, to study if the placement depended on the fluorescent protein or on the type of strain, we performed an additional biofilm experiment mixing PAO1 EYFP-tagged and PAO1 ECFP-tagged. As expected, biofilm formed by the two differently tagged wild-type strains remained mixed and no specific location or differences in the proportion of the strains were observed (Supplementary Figure S2), suggesting the absence of a hypothetical tagging effect.

Additionally, further studies were performed using ATM at 8 mg/l (fourfold PAO1 MIC). Similarly to FEP experiments, ATM did not reduce the bacterial load of biofilms formed by the resistant mutant strains while in PAO1 biofilms an important reduction of viable cells (3 log) was observed (Supplementary Figure S3). Competition experiments revealed that there was also a statistically significant increase of PAO1 bacterial load during ATM treatment when growing in mixed biofilms either with PAOΔ*mexR* or with PAOΔ*dacB* (2 and 1 log, respectively) (Supplementary Figure S3). PAO1 microcolonies formed during ATM treatment in mixed biofilms with resistant mutants were larger compared to the single biofilms (Supplementary Figure S3). Again, both resistant mutants were placed over the wild-type PAO1 acting like a shield from antibiotic molecules (Supplementary Figure S4). Furthermore, under ATM treatment, *mexR* mutation seemed to protect from filamentation and to preserve the cell shape together with the characteristic biofilm mushroom-like structure (Supplementary Figure S4).

### Resistant Mutants Failed to Protect Susceptible Populations in Planktonic Growth

To determine whether the protective effect displayed by resistant mutants could be a feature of bacterial communities regardless their mode of growth, similarly to biofilm experiments, single (every strain) and 1:1 mixed (wild-type with each resistant mutant) planktonic cultures with an initial inoculum of 10^6^ CFU/ml were carried out under FEP treatment.

**FIGURE 6:**
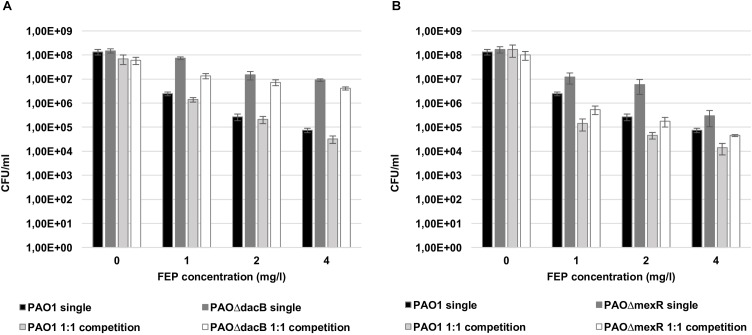
Cell counts (CFU/ml) of PAO1 and knockout mutants (**A**: PAOΔ*dacB*; **B**: PAOΔ*mexR*) single or mixed planktonic cultures (1:1 initial ratio) after 24 h of treatment with FEP (0, 1, 2, or 4 mg/l). The results represent the means (bars) and standard deviations (error bars) from at least three independent experiments.

Regarding single cultures, the bactericidal effect of FEP was higher when growing planktonically than in biofilms. FEP treatment determined a marked reduction in PAO1 bacterial load (approximately 2 log, for 1 mg/l, and 3 log for 2 and 4 mg/l of FEP) compared to control (Figure 6). The reduction in bacterial load for PAOΔ*dacB* ranged from 1 (FEP 1 and 2 mg/l) to 2 log (FEP 4 mg/l) (Figure 6A). However, the bactericidal effect was more evident for PAOΔ*mexR* decreasing from 1 (FEP 1 mg/l) to 3 log (FEP 4 mg/l) (Figure 6B).

On the contrary of what was observed in biofilm experiments, results from bacterial load data from 1:1 mixed planktonic cultures did not show a protective effect of resistant mutants on the wild-type PAO1 against antibiotic treatment (Figure 6). Indeed, total CFU numbers of resistant mutants and PAO1 were even lower in mixed cultures than in single ones (Figure 6). To test if this lack of protection was due to the low initial inoculum (10^6^ CFU/ml), further assays with 10^8^ CFU/ml were performed. However, PAO1 cell counts from 1:1 mixed cultures still under those from single cultures (Supplementary Figure S5).

## Discussion

Despite the efforts of the scientific community in recent years, understanding the whole picture of biofilm antibiotic resistance continues to be a great challenge. It is well known that the complex structure of biofilms along with the heterogeneity of bacterial cells in different physiological states may explain the increased tolerance to antibiotics (Costerton et al., 1999) but it is also imperative to focus on the genotypic diversity of bacteria coexisting in the biofilm community. Those bacteria expressing resistance mechanisms can be selected during treatments impairing the efficacy of antibiotics with the consequent failure on the eradication of the infection (Høiby et al., 2010). Nevertheless, the effect of the different resistance mechanisms on the population dynamics of the bacterial communities still remains unclear (Sorg et al., 2016). Thus, the present work studies the impact of two relevant resistance mechanisms, hyperproduction of β-lactamases and overexpression of efflux pumps, on biofilm populations during the course of β-lactam treatments, in biofilms formed by a wild type *P. aeruginosa* strain (PAO1) and its knockout (*mexR* or *dacB*) mutants.

As expected, both resistant mutants were able to survive more than PAO1 after 6 days of β-lactam treatment, confirming the important role of these classic resistance mechanisms in the biofilm mode of growth. Also, the filamentation phenomenon observed in CLSM images is consistent with the high affinity of FEP for the essential penicillin binding protein 3 (PBP3), the main target of β-lactam antibiotics (Pucci et al., 1991).

In agreement with other studies (Macià et al., 2011), our results showed that antibiotic treatment, even at sub-inhibitory concentrations, led to the selection and amplification of the resistant mutants in biofilm competition experiments. In the absence of the antibiotics, PAO1 was able to outcompete the resistant mutants. Besides the difference between initial bacterial loads, the ecological competition for space and resources led to the decrease of mutant concentration, as suggested in previous studies (Mitri et al., 2011). However, under FEP treatment PAOΔ*dacB* and PAOΔ*mexR* mutants were amplified 77 and 56 times, respectively. Accordingly, CLSM images showed the preservation of the mushroom-like structures together with an increase of the biomass of biofilms formed by resistant mutants.

Regarding the 1:1 competition experiments, surprisingly, a significant protective effect on PAO1 was observed by both resistant mutants under treatments. PAO1 bacterial load, much higher in mixed than in single biofilms (*P* < 0.05) and microscopic observations demonstrated this protective effect. The 3-D-reconstructed CLSM images revealed larger, less flattered and more organized PAO1 microcolonies in mixed biofilms (with PAOΔ*dacB* or PAOΔ*mexR*) than in single ones. Moreover, the presence of resistant mutants seemed to reduce filamentation on the susceptible subpopulations during FEP and ATM treatment, increasing evidence to their protective behavior. This structural preserving effect was also observed in a recent study using carbenicillin to treat mixed colonies of resistant and susceptible strains (Frost et al., 2018).

On the other hand, consistent with other works (Vogwill and MacLean, 2015; Frost et al., 2018) our results evidenced that there is no advantage in growing planktonically with resistant mutants since their shield effect toward susceptible populations from antibiotic molecules seems to be restricted to biofilm communities. PAO1 cell counts after treatment in mixed planktonic cultures were even lower than single cultures suggesting a strong competition between strains in mixed cultures without selection of resistance or protective effect.

Whereas our initial hypothesis, that suggested AmpC hyperproduction as an altruistic mechanism and MexAB-OprM overexpression as a selfish one, was not demonstrated, our results revealed for the first time an exciting finding; in mixed populations, mutants showing diverse resistance mechanisms protect the whole bacterial community, preserving susceptible populations from the effect of antibiotics. Interestingly, based on the microscopic observations, our results showed a pattern of genotype distribution since that both PAOΔ*dacB* and PAOΔ*mexR* were placed in the outer part of microcolonies forming a protective layer that shield susceptible population of antibiotic damage. In the case of PAOΔ*dacB*, this finding is consistent with the work of Bagge et al. (2004a) which demonstrated that the production of β-lactamase was induced in the peripheries of the microcolonies and responded to a spatial distribution. Moreover, our results are in agreement with very recent works that have shown that the presence of resistant mutants overexpressing this kind of enzyme confers a protective effect over the susceptible cells (Medaney et al., 2016; Frost et al., 2018).

While the basis for the effect of β-lactamase-mediated resistance seems clear, the explanation of the organization of PAOΔ*mexR* subpopulations on the biofilm community and moreover, how it protects the whole biofilm community is less obvious. Indeed, overexpression of efflux pumps, in mutants that expel the antibiotic molecules into the biofilm matrix is intuitively not expected to determine a major protection of the whole biofilm community. However, our results, could suggest that efflux pumps might be distributed in a way that could pump antibiotics out of the biofilm structure decreasing its internal concentration and shielding susceptible subpopulations. Some studies have evidenced that the cells producing public goods (cooperative compounds) which benefit the community can proliferate more rapidly and be placed on the edge of biofilm colonies (Driscoll and Pepper, 2010; Mitri et al., 2011; Allen et al., 2013; Nadell et al., 2016). This fact, applicable to public goods, could be extrapolated to resistance mechanisms, then those strains producing a resistance mechanism that cause a benefit to whole community would be distributed in the front part of the biofilm. In addition, the requirement of energy to hyperproduce the efflux pump systems (Poole et al., 1996; Yamaguchi et al., 2015) along with the low metabolic activity in the inner layers of biofilms (Walters et al., 2003), would support this hypothesis. Further biofilm structural and molecular analysis will be required to clarify if efflux pumps are located in a specific part of the cell membranes oriented to the exterior. Even if the absence of a definitive mechanistic explanation could be considered a limitation of this work, the phenomenum described for the first time should be a relevant start point for future studies. Moreover, future studies should confirm whether our findings using the reference strain PAO1 are broadly applicable to CF clinical isolates.

In summary, the current study demonstrated that, in biofilms, mutants showing diverse resistance mechanisms such as β-lactamase or efflux pumps hyperproduction protect the whole community, preserving wild-type susceptible populations from the effect of the antibiotics. Our results could also help to move forward understanding the role of the chronic infections frequent phenomenon of multi strain or even multi species biofilms (Burmølle et al., 2010; Ciofu et al., 2012). Moreover, our findings should be helpful to figure out why susceptible variants are not readily eradicated and are frequently isolated from CF patients despite long-term intensive antimicrobial treatments. Thus, these results represent a step forward to understand antibiotic resistance dynamics in biofilms, as well as to understand the population biology of bacterial pathogens in chronic infections, where the coexistence of susceptible and resistant variants in dense communities is a hallmark.

## Data Availability

All datasets generated for this study are included in the manuscript and/or the Supplementary Files.

## Author Contributions

ER-M, MM, and AO designed and formulated the aims and goals of the study. ER-M and MM performed the experiments. ER-M, MM, and AO analyzed the results. ER-M drafted the manuscript assisted by MM and AO.

## Conflict of Interest Statement

The authors declare that the research was conducted in the absence of any commercial or financial relationships that could be construed as a potential conflict of interest.

## Footnotes

1 http://www.eucast.org/clinical_breakpoints/
