# Supplementary figures and images for: Social Behavior of Antibiotic Resistant Mutants Within Pseudomonas aeruginosa Biofilm Communities
Source: Front Microbiol. 2019 Mar 22;10:570. doi: 10.3389/fmicb.2019.00570 (PMC6438888; doi:10.3389/fmicb.2019.00570)

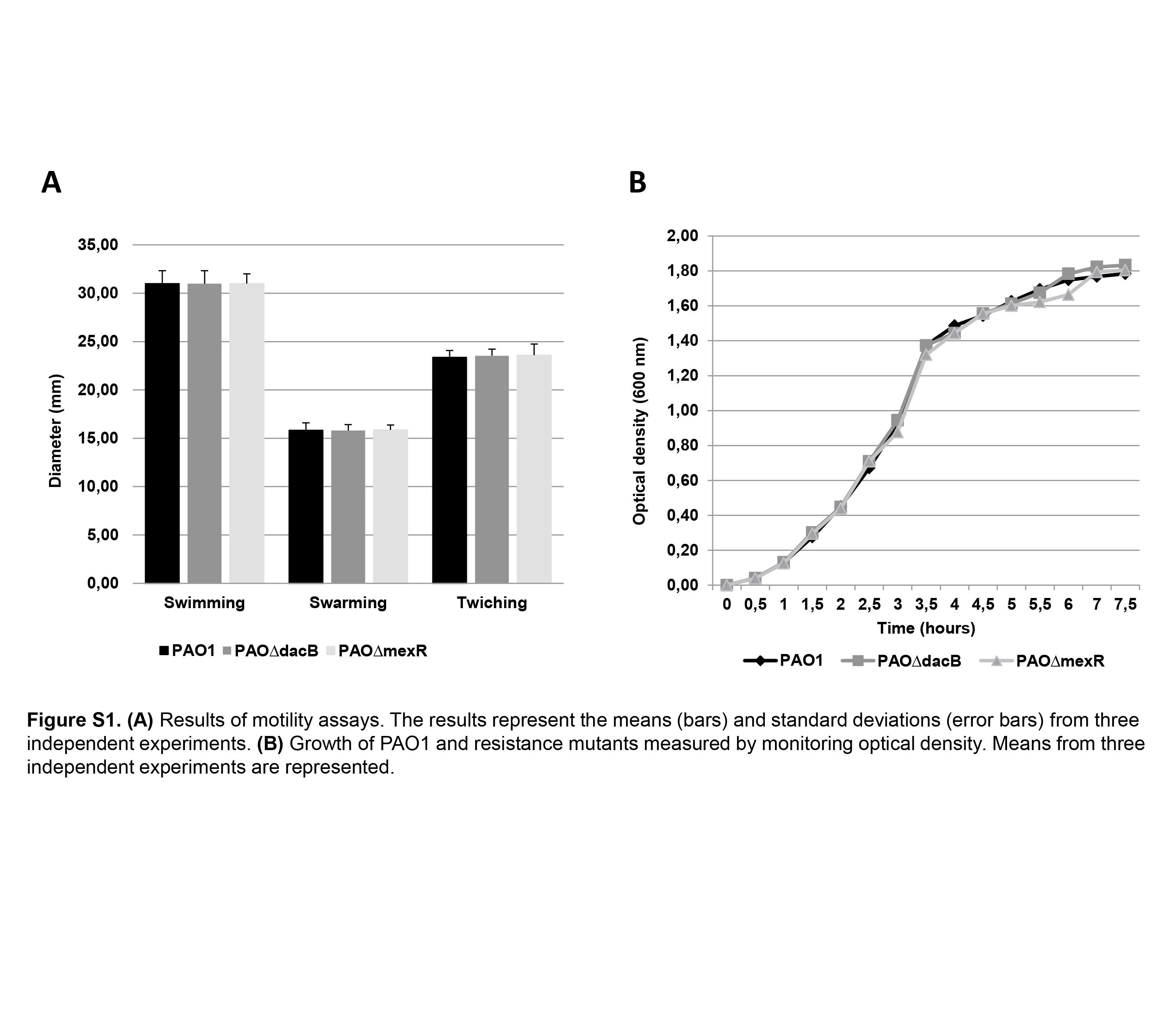

Supplement: Supplementary file 1 [file Image_1.TIF]

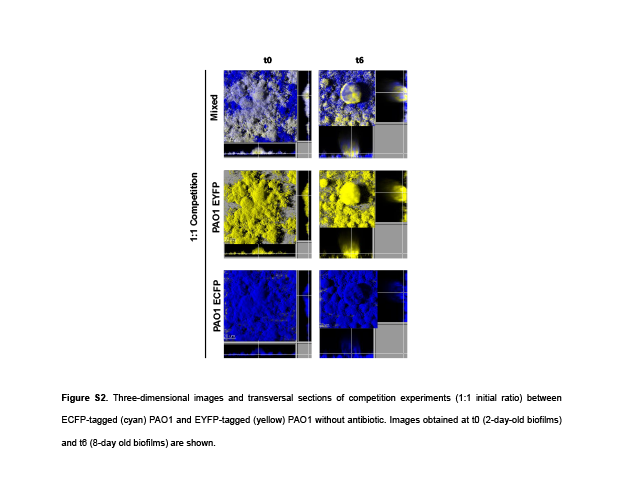

Supplement: Supplementary file 2 [file Image_2.TIF]

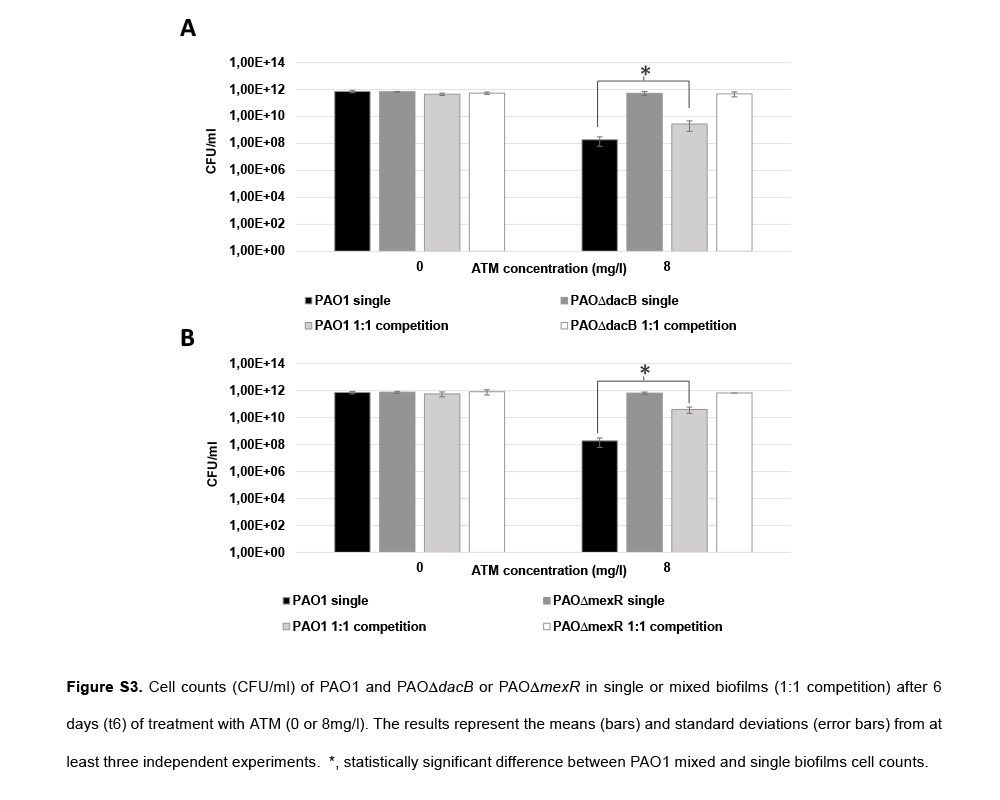

Supplement: Supplementary file 3 [file Image_3.TIF]

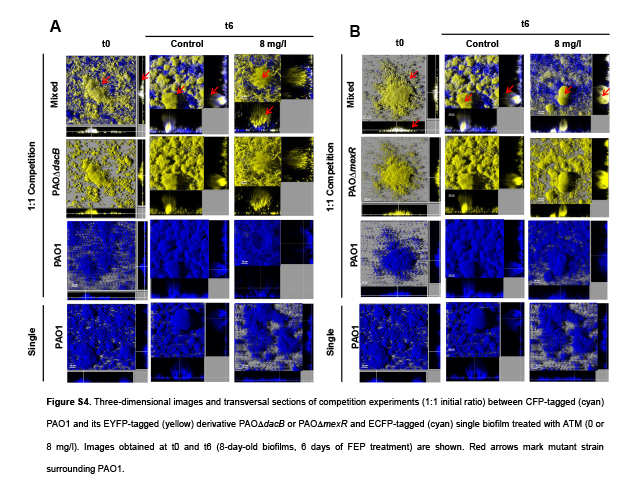

Supplement: Supplementary file 4 [file Image_4.TIF]

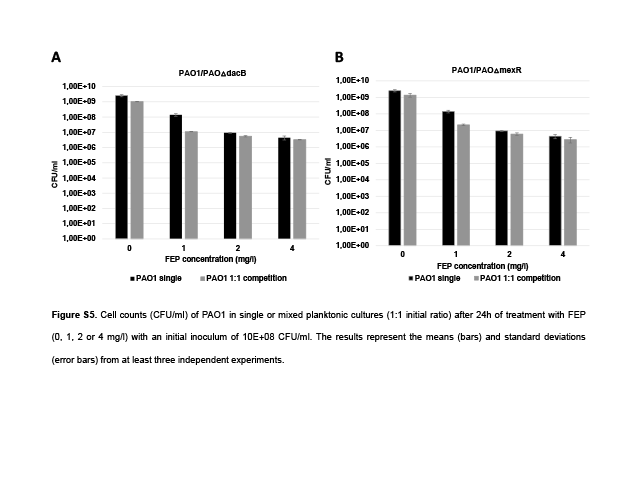

Supplement: Supplementary file 5 [file Image_5.TIF]
